# A Two-Day Continuous Nicotine Infusion Is Sufficient to Demonstrate Nicotine Withdrawal in Rats as Measured Using Intracranial Self-Stimulation

**DOI:** 10.1371/journal.pone.0144553

**Published:** 2015-12-11

**Authors:** Peter Muelken, Clare E. Schmidt, David Shelley, Laura Tally, Andrew C. Harris

**Affiliations:** 1 Minneapolis Medical Research Foundation, Minneapolis, MN, United States of America; 2 Department of Ecology, Evolution, and Behavior, University of Minnesota, Minneapolis, MN, United States of America; 3 Department of Neuroscience, University of Minnesota, Minneapolis, MN, United States of America; 4 Department of Medicine, University of Minnesota Medical School, Minneapolis, MN, United States of America; 5 Department of Psychology, University of Minnesota, Minneapolis, MN, United States of America; Universidade do Estado do Rio de Janeiro, BRAZIL

## Abstract

Avoidance of the negative affective (emotional) symptoms of nicotine withdrawal (e.g., anhedonia, anxiety) contributes to tobacco addiction. Establishing the minimal nicotine exposure conditions required to demonstrate negative affective withdrawal signs in animals, as well as understanding moderators of these conditions, could inform tobacco addiction-related research, treatment, and policy. The goal of this study was to determine the minimal duration of continuous nicotine infusion required to demonstrate nicotine withdrawal in rats as measured by elevations in intracranial self-stimulation (ICSS) thresholds (anhedonia-like behavior). Administration of the nicotinic acetylcholine receptor antagonist mecamylamine (3.0 mg/kg, s.c.) on alternate test days throughout the course of a 2-week continuous nicotine infusion (3.2 mg/kg/day via osmotic minipump) elicited elevations in ICSS thresholds beginning on the second day of infusion. Magnitude of antagonist-precipitated withdrawal did not change with further nicotine exposure and mecamylamine injections, and was similar to that observed in a positive control group receiving mecamylamine following a 14-day nicotine infusion. Expression of a significant withdrawal effect was delayed in nicotine-infused rats receiving mecamylamine on all test days rather than on alternate test days. In a separate study, rats exhibited a transient increase in ICSS thresholds following cessation of a 2-day continuous nicotine infusion (3.2 mg/kg/day). Magnitude of this spontaneous withdrawal effect was similar to that observed in rats receiving a 9-day nicotine infusion. Our findings demonstrate that rats exhibit antagonist-precipitated and spontaneous nicotine withdrawal following a 2-day continuous nicotine infusion, at least under the experimental conditions studied here. Magnitude of these effects were similar to those observed in traditional models involving more prolonged nicotine exposure. Further development of these models, including evaluation of more clinically relevant nicotine dosing regimens and other measures of nicotine withdrawal (e.g., anxiety-like behavior, somatic signs), may be useful for understanding the development of the nicotine withdrawal syndrome.

## Introduction

Cessation of tobacco use produces a nicotine withdrawal syndrome characterized by negative affect (e.g., anhedonia, anxiety), increased appetite/weight gain, cognitive deficits, and somatic symptoms (e.g., gastrointestinal discomfort) [1–5]. Avoidance of symptoms of nicotine withdrawal, particularly those related to negative affect, is one factor that contributes to tobacco addiction [1–3, 6, 7]. For example, duration and severity of negative affective withdrawal symptoms are robust predictors of relapse in abstinent smokers [8–11]. Elucidating the behavioral and neurobiological mechanisms contributing to the negative affective component of nicotine withdrawal is therefore essential for developing more effective treatments for smoking cessation.

Animal models have been useful for studying nicotine withdrawal. Rodents exhibit a nicotine withdrawal syndrome following abrupt cessation of chronic nicotine exposure (spontaneous withdrawal) or administration of a nicotinic acetylcholine receptor (nAChR) antagonist during chronic nicotine exposure (antagonist-precipitated withdrawal) [4–7, 12]. This nicotine withdrawal syndrome includes somatic signs such as abdominal constrictions and cheek tremors, as well as behavioral effects including suppression of operant responding for food, conditioned place aversion, and elevations in the minimal (threshold) current that maintains intracranial self-stimulation (ICSS). Some of these behavioral measures (e.g., ICSS threshold elevations, a putative measure of anhedonia) are thought to model the negative affective component of nicotine withdrawal that plays a particularly important role in tobacco addiction [2, 13, 14].

Establishing the minimal nicotine exposure conditions required to elicit nicotine withdrawal in animals, as well as understanding moderators of these conditions, would provide critical insights into the processes mediating the development of the nicotine withdrawal syndrome. These processes may represent particularly important targets for nicotine withdrawal-related research and treatment. Supporting the clinical relevance of early nicotine withdrawal symptoms, severity of withdrawal following limited tobacco use predicted the progression to daily smoking and vulnerability to relapse in adolescents [15, 16, 17],

To the extent that findings from preclinical models of nicotine withdrawal translate to human smokers, understanding the development of nicotine withdrawal could also inform the regulation of tobacco products by the Food and Drug Administration (FDA). Fundamental to effective FDA tobacco control policy is establishment of the lowest levels of nicotine exposure that support the development of tobacco addiction, as well as characterization of biological and behavioral determinants of “nicotine addiction thresholds” [18–22]. To date, preclinical work on this topic has focused on understanding moderators of thresholds for nicotine reinforcement as measured by i.v. nicotine self-administration [21–24]. Understanding determinants of the threshold nicotine exposure conditions for demonstrating withdrawal in animals would complement this work and provide additional scientific information to support FDA regulatory efforts.

The minimal levels of nicotine exposure required to demonstrate nicotine withdrawal in animals have not been well established. Most studies in rats involve at least 6–7 days of a continuous nicotine infusion via osmotic minipump (3.0–3.2 mg/kg/day) prior to assessment of withdrawal [4, 12, 25, 26], and it is often assumed that these nicotine exposure conditions are necessary to observe withdrawal signs in rats [4, 27, 28]. However, Vann et al. [29] found that the nAChR antagonist mecamylamine precipitated suppression of operant responding for food following 4 days, but not 3 days, of a continuous nicotine infusion via osmotic minipump (3.0 mg/kg/day). In addition, withdrawal-induced suppression of operant responding in rats was reported following 3–4 daily acute nicotine injections (0.1–0.4 mg/kg, s.c.) [30, 31]. We also found that mecamylamine elicited increases in ICSS thresholds and somatic signs in rats following a single acute nicotine injection (0.5 mg/kg, s.c.) [32].

The latter findings suggest that ICSS may be a particularly sensitive measure of the early development of nicotine withdrawal, and support the utility of this assay for further research in this area. A further advantage of ICSS is that it has considerable predictive validity as a measure of the negative affective component of nicotine withdrawal [26, 33, 34]. In addition, the ICSS threshold procedure used in the current studies is relatively insensitive to response rate, thereby providing a valid measure of the reinforcing effects of the brain stimulation even in the presence of treatments that inhibit motor function [35, 36]. Finally, because there is little or no satiation to the reinforcing effects of electrical brain stimulation, ICSS can be measured repeatedly within-subjects without loss of sensitivity [25, 32, 37].

The goal of this study was to evaluate the minimal duration of continuous nicotine infusion required to demonstrate nicotine withdrawal in rats as measured by elevations in ICSS thresholds. While the ability of a relatively short-term continuous nicotine infusion to elicit withdrawal in rats was previously studied using suppression of operant responding for food as a dependent measure [29], examining this issue using ICSS is important given the unique advantages provided by this assay. Experiment 1 evaluated the ability of mecamylamine to precipitate increases in ICSS thresholds when administered every 1–2 test days throughout the course of a 2-week continuous nicotine infusion. Because a 2-day continuous infusion was sufficient to demonstrate antagonist-precipitated withdrawal in Experiment 1, Experiment 2 evaluated whether abrupt cessation of this duration of nicotine infusion could elicit spontaneous withdrawal. As a positive control, we also evaluated antagonist-precipitated withdrawal (Experiment 1) or spontaneous withdrawal (Experiment 2) in traditional models involving more prolonged nicotine exposure (i.e., 14 or 9 days of continuous infusion) prior to withdrawal testing.

## Materials and Methods

### Animals

Male Wistar rats (Charles River Laboratories, Wilmington, MA) weighing 275–300 g upon arrival were individually housed in a colony room with unlimited access to food and water. Rats were housed under a reversed 12-hour light/dark cycle and were tested during the dark (active) phase. Animals were given at least one week to acclimate to the experimental housing following arrival in the colony. Animal husbandry and experimental protocols were approved by the Institutional Animal Care and Use Committee of the Minneapolis Medical Research Foundation (protocol # 08-08R) in accordance with the 2011 NIH Guide for the Care and Use of Laboratory Animals and the Guidelines for the Care and Use of Mammals in Neuroscience and Behavioral Research (National Research Council 2003). No animals were euthanized as part of these studies. All efforts were made to minimize animal suffering.

### Drugs

Nicotine bitartrate and mecamylamine hydrochloride (Sigma Chemical Co., St. Louis, MO) were dissolved in sterile saline. The pH of the nicotine solution was adjusted to 7.4 with dilute NaOH. Nicotine doses are expressed as the base. Nicotine was administered via osmotic minipump (see below). Mecamylamine was administered via s.c. injection at a volume of 1.0 ml/kg.

### Osmotic minipump surgery

Rats were anesthetized with an isoflurane/oxygen vapor mixture (1–3% isoflurane) and prepared with Alzet osmotic minipumps (model 2ML2; Durect Corporation, Cupertino, CA) as described previously [36, 38]. Pumps were filled with physiological saline or nicotine solution adjusted to deliver a nicotine dose of 3.2 mg/kg/day, and were primed in saline for 1 hour prior to implantation under the skin. Because the osmotic pumps have a start-up time of 4–6 hours (www.alzet.com/downloads/2ML2specs.pdf), delivery of nicotine actually began 3–5 hours after osmotic pump implantation. Rats received injections of the analgesic buprenorphine (0.1 mg/kg, s.c.) and the antibiotic ceftriaxone (5.25 mg, i.m.) immediately following surgery and again 24 hr later (after that day’s behavioral test).

### Intracranial self-stimulation

The surgery, apparatus, and training procedure used here are described in detail elsewhere [26, 38, 39]. Briefly, animals were anesthetized with i.m. ketamine (75 mg/kg) / xylazine (7.5 mg/kg) and implanted with a bipolar stainless steel electrode in the medial forebrain bundle at the level of the lateral hypothalamus. Animals were later trained to respond for electrical brain stimulation (0.1 ms cathodal squarewave pulses at a frequency of 100 Hz for 500 ms) by rotating a metal wheel manipulandum affixed to the front wall of an operant chamber. Following acquisition of robust responding for brain stimulation under an FR1 schedule, rats were trained on a discrete-trial current-threshold procedure as described previously [26, 35, 38]. Each trial was initiated with presentation of a non-contingent electrical stimulus followed by a 7.5-second window during which a positive response on the wheel manipulandum produced a second, contingent stimulation identical to the first. Lack of responding in the 7.5-second time window was considered a negative response. Each positive or negative response was followed by a variable inter-trial interval averaging 10 seconds (range = 7.5 to 12.5 seconds), during which time additional responses delayed onset of the subsequent trial by 12.5 seconds. Stimulus intensities were presented in four alternating descending and ascending series (step size = 5 uA), with five trials presented at each current intensity step. The current threshold for each series was defined as the midpoint between two consecutive current intensity steps that yielded three or more positive responses and two consecutive current intensity steps that yielded three or more negative responses. The overall threshold for the ≈ 45 minute session was defined as the mean of the current thresholds from the four alternating series, and represents a measure of the reinforcing effects of the brain stimulation. Response latencies were defined as the time between onset of the non-contingent stimulus and the animal’s response on the wheel manipulandum, averaged across all trials in which a positive response was made. Response latencies represent a sensitive measure of general motor function, as treatments that interfere with performance of the task (e.g., drugs with sedative effects) reliably increase response latencies [35, 36, 40].

### Experiment 1: Mecamylamine-precipitated nicotine withdrawal

Rats were tested for ICSS in twice daily sessions conducted at 8:00 and 11:00 AM Monday through Friday until ICSS thresholds were stable (<10% coefficient of variation over a five day period with no apparent trend). To habituate animals to the injection procedure, saline was administered 10 minutes prior to the second test for at least 5 days and until thresholds were stable. Following the second test on the final day of the baseline period (always a Monday), rats satisfying the above stability criteria were implanted with an osmotic pump delivering saline or nicotine at a rate (3.2 mg/kg/day) that is commonly used to study nicotine withdrawal in rats [25, 32, 36, 38]. The following day (i.e., test day 1), rats were tested for ICSS at 8:00 AM (pre-test) and 11:00 AM (post-test) as during baseline, but received either saline or 3.0 mg/kg mecamylamine prior to the post-test depending on group assignment (see below). This procedure was repeated daily throughout the course of the 14-day infusion, resulting in a total of 10 experimental test days due to weekend breaks in testing. These weekend breaks, which occurred between test days 4–5 and test days 9–10, had no apparent effect on the outcomes (see Results).

**Fig 1:**
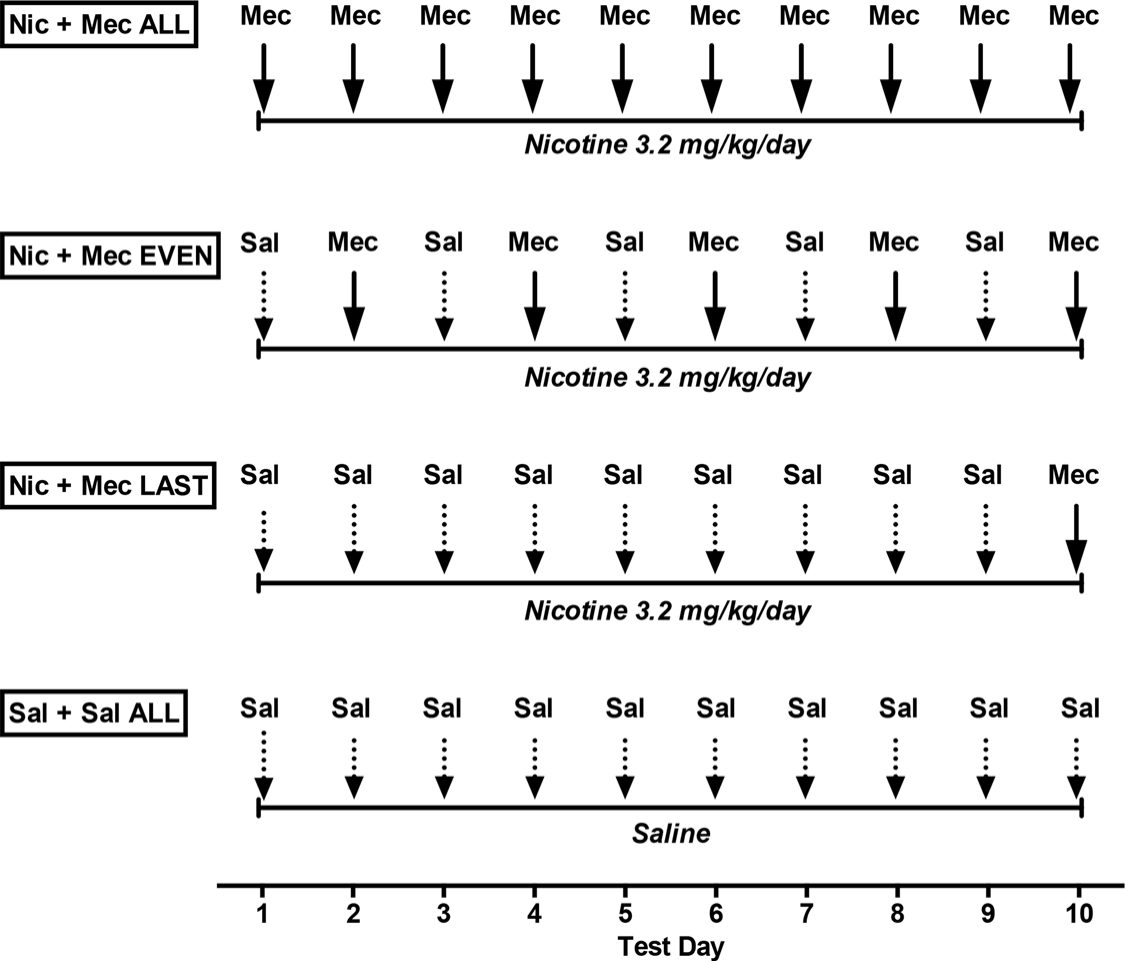
Procedural timeline for Experiment 1. Mec = 3.0 mg/kg mecamylamine. Sal = Saline. Chronic infusion condition is italicized. See text for further details.

Continuous infusion and acute injection conditions for each of the 4 experimental groups are shown in Fig 1. Rats in the Nic + Mec ALL group (n = 6) were infused with nicotine and received 3.0 mg/kg mecamylamine 10 minutes prior to the post-test on each of the 10 test days. This mecamylamine dose was used because it reliably precipitates robust elevations in ICSS thresholds following at least 6–7 days of a continuous infusion of this nicotine dose, but does not affect ICSS thresholds in saline-infused rats [32, 41, 42, 43]. This mecamylamine dose, but not a lower dose (1.5 mg/kg), also elicited elevations in ICSS thresholds when administered following a single nicotine injection [32], supporting its utility for eliciting withdrawal effects after minimal nicotine exposure. Pilot studies suggested that the schedule of mecamylamine injections used in the Nic + Mec ALL group could impede the development of nicotine withdrawal (data not shown). To examine the effects of a less intense mecamylamine injection regimen, rats in the Nic + Mec EVEN group (n = 7) were treated as described for the Nic + Mec ALL group except that animals received saline on odd-numbered test days and mecamylamine on even-numbered test days, such that mecamylamine was first administered following 2 days of the infusion. Rats in the Nic + Mec FINAL group (positive control, n = 8) were infused with nicotine and received saline prior to the post-test on each of test days 1–9 and mecamylamine prior to the post-test on the final (10^th^) test day, such that mecamylamine was first administered following 14 days of continuous nicotine infusion. Rats in the Sal + Sal ALL group (negative control, n = 9) received a continuous infusion of saline and were injected with saline 10 minutes prior to the post-test on all test days.

### Experiment 2: Spontaneous nicotine withdrawal

A separate set of rats was tested for ICSS in once-daily sessions conducted Monday-Friday until thresholds were stable, at which rats were implanted with osmotic pumps delivering saline or 3.2 mg/kg/day nicotine. For two groups, pumps were implanted on a Monday and removed on the Wednesday of the same week, resulting in a 2-day infusion of saline (2-day Sal, n = 8) or nicotine (2-day Nic, n = 6). For the other two groups, pumps were implanted on a Monday and removed on the Wednesday of the following week, resulting in a 9-day infusion of saline (9-day Sal, n = 6) or nicotine (9-day Nic, n = 6). Rats continued to be tested for ICSS in daily sessions following osmotic pump implantation. To evaluate spontaneous withdrawal, rats were tested for ICSS on the Thursday and Friday following pump removal (i.e., 20 and 44 hours after pump explantation, respectively), as well as on the following Monday (i.e., 116 hours following pump explantation). These time points were chosen based on the time course of elevations in ICSS thresholds during spontaneous nicotine withdrawal reported by our lab and others [26, 43, 44].

### Statistical Analyses

In Experiment 1, ICSS thresholds (in μA) and response latencies (in seconds) during pre- and post-tests were expressed as a percentage of baseline (i.e., mean during the last 5 sessions prior to osmotic pump implantation). ICSS threshold and latency data were analyzed using separate three-factor ANOVAs with group as a between-subject factor and test session (i.e., pre-test versus post-test) and test day as within-subject factors. ICSS threshold and latency data for each test session were subsequently analyzed using separate two-factor (group x test day) ANOVAs followed by Tukey post hoc tests comparing groups on each test day. In groups receiving mecamylamine, Pearson's correlation analysis was used to evaluate the relationship between the effects of mecamylamine on ICSS thresholds and latencies during post-tests. In Experiment 2, ICSS threshold and latency data were expressed as a percentage of baseline (i.e., mean during the last 5 sessions prior to osmotic pump removal) and evaluated using separate two-factor (group x time point) ANOVAs, followed by Tukey post hoc tests comparing groups at each time point. Statistical significance for all analyses was set at p < 0.05.

## Results

### Experiment 1: Mecamylamine-precipitated nicotine withdrawal

#### ICSS thresholds and latencies during baseline sessions

ICSS thresholds and response latencies did not differ significantly between groups during baseline sessions for either pre- or post-injection tests (Table 1). The greater variability in baseline thresholds for the Nic + Mec ALL group is due to a single animal with a baseline threshold of 214.1 μA. This relatively high baseline value is within the range typically observed in our lab, and is controlled for in the analysis by expressing data as percentage of baseline.

**Table 1:** Baseline measures for Experiment 1. Mean (±SEM) ICSS thresholds (in μA) and response latencies (in seconds) during baseline sessions for pre- and post- tests in Experiment 1.

	Pre-test Baseline		Post-test Baseline	
	Thresholds	Latencies	Thresholds	Latencies
**Nic + Mec ALL**	112.5 ± 25.8	2.5 ± 0.1	115.2 ± 26.5	2.4 ± 0.1
**Nic + Mec EVEN**	85.6 ± 6.1	2.5 ± 0.1	87.9 ± 6.7	2.5 ± 0.2
**Nic + Mec FINAL**	93.8 ± 9.5	2.4 ± 0.2	94.1 ± 8.8	2.4 ± 0.2
**Sal + Sal ALL**	88.5 ± 4.5	2.3 ± 0.2	91.1 ± 3.7	2.3 ± 0.2

#### ICSS thresholds during pre- and post-tests

Three-factor ANOVA indicated significant effects of group (*F*(3,26) = 4.7, *p* <0.01), test session (i.e., pre- versus post-test) (*F*(1,26) = 46.1, *p* <0.0001), and test day (*F*(9,234) = 5.5, *p* <0.0001) on ICSS thresholds. There were also significant group x test session (*F*(3,26) = 12.3, *p* <0.0001), group x test day (*F*(27,234) = 4.9, *p* <0.0001), and test session x test day *(F*(9,234) = 10.7, *p* <0.0001) interactions, as well as a significant group x test session x test day interaction (*F*(27,234) = 7.2, *p* <0.0001).

**Fig 2:**
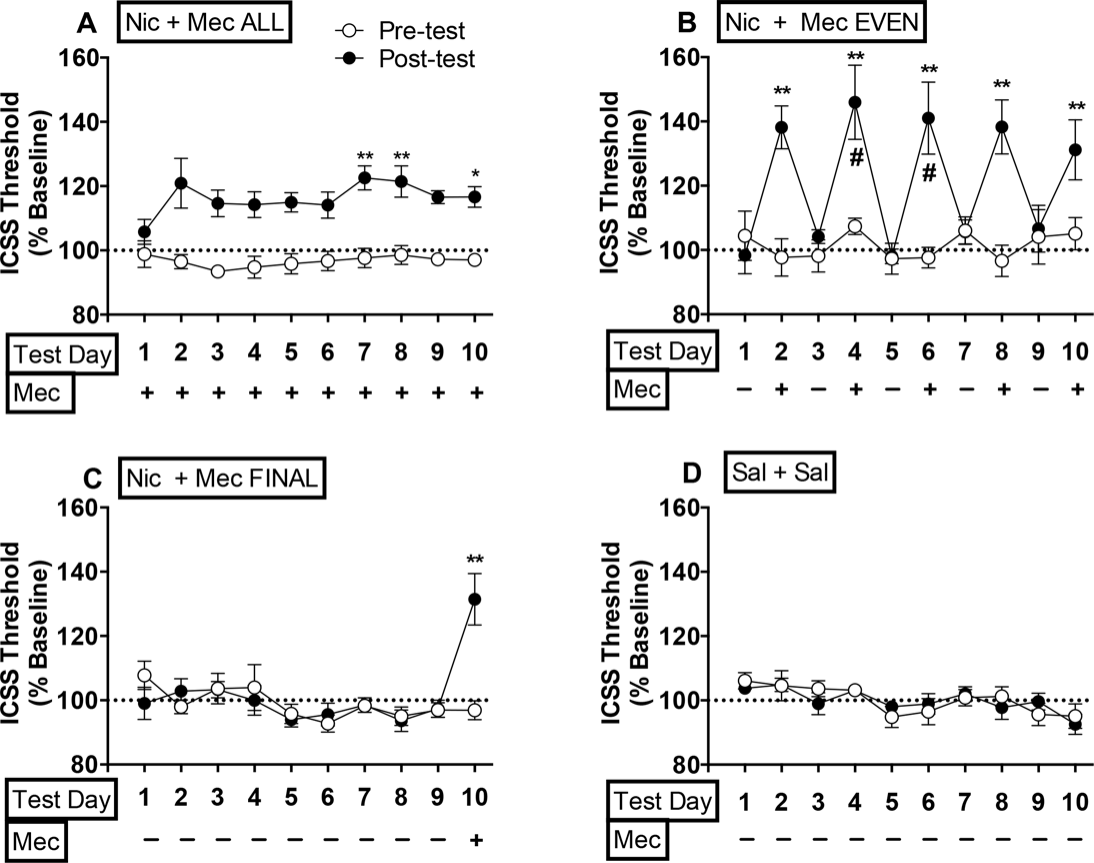
Mecamylamine elicits elevations in ICSS thresholds in rats receiving a continuous nicotine infusion for 2 or more days. Mean (±SEM) ICSS thresholds (expressed as percent of baseline) during pre- and post-tests in Experiment 1. Mecamylamine (+) or saline (-) was administered prior to post-tests. See Materials and Methods section for definition of group abbreviations. *^,^** Different from Sal + Sal ALL group on that test day, p <0.05 or 0.01. # Nic + Mec EVEN group elevated compared to Nic + Mec ALL group on that test day, *p* < 0.01.

The above analysis indicates that group, test session, and test day all impacted ICSS thresholds, but that the nature of these effects was dependent on the other factors. To further explore this three-way interaction, we evaluated the effects of group and test day on data within each test session (i.e., pre-tests or post-tests). Two-factor ANOVA on threshold data during pre-tests indicated a significant effect of test day (*F*(9,234) = 3.1, *p* <0.0001), reflecting a modest (≤ 10–15%) reduction in ICSS thresholds across pre-tests for all groups, but no effect of group or group x test day interaction (see Fig 2A–2D). These data indicate that any between-group differences in ICSS thresholds during post-tests (see below) were due to drug treatment.

During post-tests, there were significant effects of group (*F*(3,26) = 12.3, *p* <0.0001), test day (*F*(9, 234) = 9.9, *p* <0.0001), and a significant group x test day interaction (*F*(27,234) = 8.2, *p* <0.0001). ICSS thresholds during post-tests therefore varied as a function of both group and test day. Thresholds in the Nic + Mec ALL group were clearly elevated during the post-test compared to the pre-test beginning on test day 2 and continuing throughout the duration of the infusion (see Fig 2A). However, thresholds in this group only differed from those in the Sal + Sal ALL group (shown in Fig 2D) during test days 7, 8, and 10 (q = 4.2–4.9, *p* <0.05 or 0.01; see Fig 2A). In contrast, thresholds were elevated in the Nic + Mec EVEN group compared to the Sal + Sal ALL group on all test days in which mecamylamine was administered (i.e., even-numbered test days), including session 2 (q = 7.2–9.1, *p* <0.01; Fig 2B). The effects of mecamylamine were generally greater in the Nic + Mec EVEN group compared to the Nic + Mec ALL group, a difference that was significant on test days 4 and 6 (q = 6.2 and 5.2, respectively, *p* < 0.01) and marginally significant on test day 2 and 8 (q = 3.4 and 3.3, respectively, *p* = 0.085 and 0.095). Thresholds in the Nic + Mec LAST group were elevated compared to the Sal + Sal ALL group on the single test day on which mecamylamine was administered (i.e., test day 10; t = 8.7, p <0.01; Fig 2C). Magnitude of this withdrawal effect was similar to that observed in the Nic + Mec EVEN group on all even-numbered test days, including test day 2 (compare Fig 2B and 2C).

#### ICSS latencies during pre- and post-tests

Three-factor ANOVA indicated significant effects of group (*F*(3,26) = 3.4, *p* <0.05), test session (*F*(1,26) = 20.8, *p* <0.0001), and test day (*F*(9,234) = 2.3, *p* <0.05) on response latencies, as well as significant group x test session (*F*(3,26) = 11.1, *p* <0.0001), group x test day (*F*(27,234) = 2.2, *p* <0.01), test session x test day (*F*(9,234) = 4.4, *p* <0.0001) and group x test session x test day (*F*(27,234) = 3.2, *p* <0.0001) interactions.

**Fig 3:**
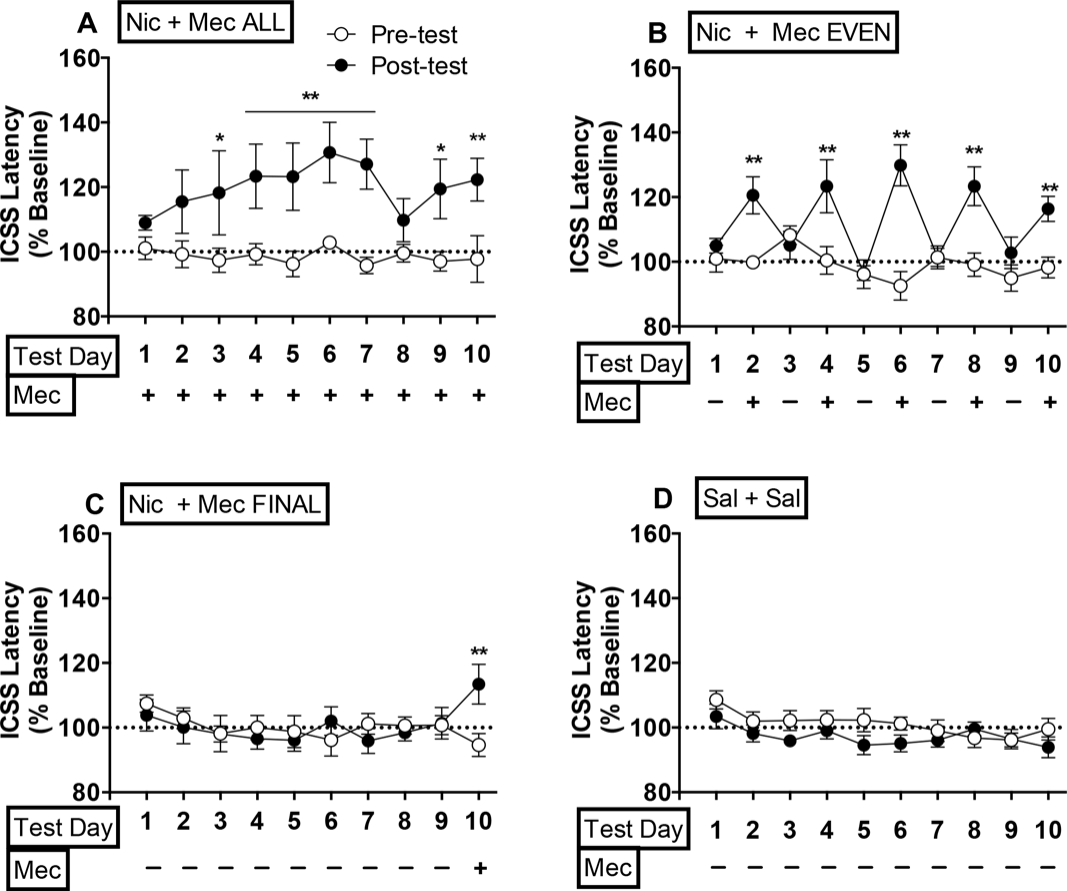
Mecamylamine elicits elevations in ICSS latencies in rats receiving a continuous nicotine infusion for 2 or more days. Mean (±SEM) ICSS latencies (expressed as percent of baseline) during pre- and post-tests in Experiment 1. *^,^** Different from Sal + Sal ALL group on that test day, *p* <0.05 or 0.01.

To further explore the three-way interaction between factors, we evaluated the effects of group and test day on data within each test session (i.e., pre-tests or post-tests). There was a significant effect of test day on ICSS latencies during pre-tests (*F*(9,234) = 2.1, *p* <0.05), reflecting a modest reduction in ICSS latencies across pre-tests, but no effect of group or group x test day interaction (see Fig 3A–3D).

During post-tests, there were significant effects of group (*F*(3,26) = 7.7, *p* <0.001), test day (*F*(9, 234) = 4.1, *p* <0.0001), and a significant group x test day interaction (*F*(27,234) = 3.8, *p* <0.0001). Latencies were significantly elevated in the Nic + Mec ALL group compared to the Sal + Sal ALL group on test days 3–7, 9, and 10 (q = 4.3–6.9, p <0.05 or 0.01; Fig 3A). Mecamylamine elevated latencies in the Nic + Mec EVEN group compared to the Sal + Sal ALL group on all test days in which it was administered (i.e., even-numbered test days) (q = 4.5–7.0, *p* <0.01) (Fig 3B). Thresholds in the Nic + Mec LAST group were elevated compared to those in the Sal + Sal ALL group only when mecamylamine was administered during session 10 (q = 4.1, *p* <0.05; Fig 3C).

#### Relationship between ICSS thresholds and latencies during post-tests

Mecamylamine significantly elevated both ICSS thresholds and response latencies (see Figs 2 and 3). This raises the possibility that mecamylamine’s inhibition of the reinforcing effects of the brain stimulation, indicated by the threshold data, were secondary to its motor suppressive effects, indicated by the latency data (see Materials and Methods for further description of these ICSS measures). However, magnitude of mecamylamine’s effects on ICSS thresholds and latencies were not correlated on any test day in any group (See S1 Table), suggesting that effects of mecamylamine on these measures of ICSS were independent.

### Experiment 2: Spontaneous nicotine withdrawal

#### ICSS thresholds and latencies during baseline sessions

ICSS thresholds and response latencies did not differ between groups during baseline sessions (Table 2).

**Table 2:** Baseline measures for Experiment 2. Mean (±SEM) ICSS thresholds (in μA) and response latencies (in seconds) during baseline sessions in Experiment 2.

	Thresholds	Latencies
**2-day Nic**	111.0 ± 24.5	2.5 ± 0.1
**2-day Sal**	90.1 ± 4.9	2.7 ± 0.2
**9-day Nic**	105.0 ± 15.1	2.3 ± 0.1
**9-day Sal**	101.8 ± 7.4	2.6 ± 0.1

#### ICSS thresholds and latencies during test sessions

**Fig 4:**
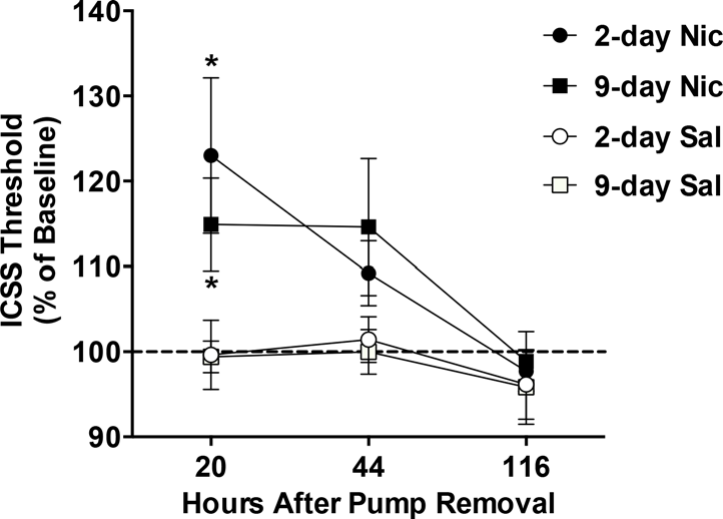
Cessation of a 2-day or 9-day continuous nicotine infusion elicits elevations in ICSS thresholds. Mean (±SEM) ICSS thresholds (expressed as percent of baseline) following osmotic pump removal in Experiment 2. See Materials and Methods section for definition of group abbreviations. * 2- or 9-day Nic group different from its respective Sal control group at that time point, p <0.05.

Two-factor ANOVA indicated significant effects of group (*F*(3,22) = 4.2, *p* <0.05) and time point (*F*(2,44) = 8.2, *p* < 0.001) on ICSS thresholds. Thresholds were elevated in the 2-day Nic group and the 9-day Nic group compared to their respective saline control groups 20 hours after osmotic pump removal (q = 5.1 and 4.8, respectively, *p* < 0.05; Fig 4), but these groups did not differ from each other. There were no significant differences between groups at subsequent time points (Fig 4). There was also no significant effect of group, time point, or group x time point interaction on response latencies (all p-values > 0.05; see S2 Table).

## Discussion

These studies evaluated the minimal duration of continuous nicotine infusion required to demonstrate nicotine withdrawal in rats as measured by elevations in ICSS thresholds, a measure of anhedonia-like behavior. In Experiment 1, administration of the nAChR antagonist mecamylamine on alternate test days throughout the course of a 2-week nicotine infusion elicited ICSS threshold elevations beginning on the second day of the infusion. Magnitude of withdrawal in the Nic + Mec EVEN group did not change with further nicotine exposure and mecamylamine injections, and was similar to that observed in a positive control group (Nic + Mec LAST) first administered mecamylamine following a more prolonged (14-day) nicotine infusion. Expression of a significant withdrawal effect was delayed in nicotine-infused rats receiving mecamylamine on all test days rather than on alternate test days (i.e., Nic + Mec ALL group). In Experiment 2, rats exhibited a transient increase in ICSS thresholds following cessation of a 2-day continuous nicotine infusion. Magnitude of this spontaneous withdrawal effect was similar to that observed in a positive control group receiving a 9-day nicotine infusion.

These findings suggest that a 2-day continuous nicotine infusion (3.2 mg/kg/day) can reliably elicit nicotine withdrawal in rats, at least under the experimental conditions established here. Our data contrast with a report that mecamylamine failed to precipitate suppression of operant responding for food in rats following a 3-day continuous infusion of nicotine (3.0 mg/kg) [29]. This discrepancy raises the possibility that ICSS is a more sensitive measure of the early development of nicotine withdrawal than suppression of operant responding. Alternatively, it could reflect other methodological differences across studies including nicotine dose, rat strain, and feeding/housing conditions. Regardless, mecamylamine-precipitated withdrawal was observed in [29] following a 4-day continuous nicotine infusion. As such, both data sets suggest that the duration of continuous nicotine infusion required to demonstrate nicotine withdrawal in rats is shorter than the duration traditionally used (i.e., at least 6–7 days).

Our findings suggest that, once established following a 2-day infusion, nicotine withdrawal tested under the current conditions is minimally impacted by further increases in duration of infusion (Experiments 1 and 2) or by the assessment of repeated daily withdrawal episodes (Experiment 1). In contrast, increases in infusion duration and repeated withdrawal episodes can progressively exacerbate severity of withdrawal from other drugs such as alcohol or morphine [45–48]. Our data do, however, complement previous findings in rats indicating no effect, or only a limited effect, of infusion duration or repeated withdrawal episodes on severity of nicotine withdrawal [29, 32, 44, 49]. For example, there was no tolerance or sensitization of ICSS threshold elevations or somatic signs during repeated antagonist-precipitated withdrawal in rats receiving a chronic nicotine infusion [44] or repeated acute nicotine injections [32].

The above findings support the notion that severity of nicotine withdrawal may quickly reach an asymptotic level once minimal nicotine exposure conditions are achieved [44, 50], an account supported by some human studies [51, 52]. On the other hand, some studies in mice [53, 54] and humans [55, 56] have reported a progressive increase in withdrawal severity as a function of duration of nicotine exposure and/or number of withdrawal episodes. In fact, escalation in withdrawal severity during early tobacco use has been proposed to play a key role in the development of tobacco addiction in adolescents [15, 16, 57]. Further study of the role of duration of nicotine exposure and number of withdrawal episodes in the development of the nicotine withdrawal syndrome in animals and humans is warranted.

Our data raise the possibility that the neurobiological adaptations underlying nicotine withdrawal are fully developed following a 2-day continuous nicotine infusion, at least as measured using ICSS. Alternatively, different mechanisms may underlie nicotine withdrawal following a 2-day infusion versus longer durations, despite the similar magnitude of withdrawal observed across these conditions. This issue could be addressed by comparing our findings with the time course of the development of neurobiological changes implicated in nicotine withdrawal during a continuous nicotine infusion. For example, nicotine exposure produces an increase in nAChR binding sites (i.e., upregulation) in numerous brain regions including the ventral tegmental area, nucleus accumbens, and hippocampus [58–62], a phenomenon that has been linked to nicotine withdrawal [7, 63–66]. The time course of nAChR upregulation during a chronic nicotine infusion has not been well established. Nguyen and colleagues [67] observed significant nAChR upregulation in cerebral cortex, superior colliculus, and thalamus following 14 days, but not 16 hours, of a continuous nicotine infusion (6.0 mg/kg/day) [67], but did not study intermediate time points. Gould and colleagues [68] reported nAChR upregulation in dorsal hippocampus and expression of nicotine withdrawal-induced deficits in learning in mice following a 4-day continuous nicotine infusion (6.3 mg/kg/day), but not following infusion durations of 1, 2, or 3 days. However, the relevance of the latter data to our findings is unclear given the different species used across studies. Further characterization of the time course of nAChR upregulation and other adaptations (e.g., suppression of mesolimbic dopamine levels [4, 69]) in withdrawal-related brain regions during a chronic nicotine infusion is clearly needed.

The higher frequency of mecamylamine injections in the Nic + Mec ALL group in Experiment 1 appeared to attenuate the development of nicotine withdrawal. Of potential relevance, Vann et al. [29] found that the development of nicotine withdrawal typically induced by a cumulative 4-day continuous nicotine infusion could be prevented by interrupting the infusion for a 24 hour period. Given that mecamylamine has a half-life of ≈ 1 hour in rats [70], the disruption of nicotine’s effects following each mecamylamine injection in our study would clearly be of a shorter duration than the 24 hour nicotine-free period used in [29]. Nonetheless, both data sets emphasize the importance of continued stimulation of nAChRs in the induction of nicotine withdrawal. These findings complement human studies showing that development of nicotine withdrawal is greater under conditions of regular rather than intermittent smoking [55, 71], and implicate pattern of nicotine exposure as a key determinant of the development of the nicotine withdrawal syndrome.

We previously found that 3.0 mg/kg mecamylamine elevated ICSS thresholds when administered 2 hours after only a single nicotine injection (0.5 mg/kg, s.c.) [32]. The inability of the same mecamylamine dose to precipitate withdrawal in the Nic + Mec ALL group following a 1-day nicotine infusion in Experiment 1 may therefore be surprising. However, it is well established that the neurobiological and behavioral consequences of nicotine can differ considerably when it is administered as a continuous infusion versus a rapid bolus [72, 73, 74]. Furthermore, the acute nicotine dose used in Harris et al. (2013) would almost certainly produce nicotine serum levels higher than those achieved via osmotic minipump infusion in this study [75–78]. Other methodological factors unique to the current protocol (e.g., surgical implantation of osmotic minipumps) may also have inhibited the expression of withdrawal following a 1-day continuous nicotine infusion.

We elected to administer nicotine continuously via osmotic minipump (3.2 mg/kg/day) because this is the most commonly used approach for demonstrating nicotine withdrawal in rats [12]. This exposure regimen also has some clinical relevance in that it produces nicotine serum levels within the range of those observed in heavy smokers (≈ 40–50 ng/ml) [12, 77–80]. Nonetheless, it differs from nicotine exposure in humans in other respects including total daily nicotine dose (i.e., 3.2 mg/kg/day versus 0.14–1.14 mg/kg/day [81]), route and pattern of administration (infused and continuous versus inhaled and intermittent), and contingency of nicotine exposure (i.e., non-contingent versus contingent). All of these variables can impact the behavioral effects of nicotine [82–85]. Characterization of the early development of nicotine withdrawal using nicotine dosing regimens that more accurately simulate nicotine exposure in humans, such as those involving nicotine inhalation, bolus dosing, and/or contingent nicotine exposure, is needed to confirm the clinical relevance of our findings.

A potential limitation of Experiment 1 is that effects of mecamylamine in saline-infused animals were not studied, raising the possibility that mecamylamine’s effects in nicotine-dependent animals were non-specific. However, it is well established in our laboratory and others that single or repeated injection of this dose of mecamylamine does not affect ICSS thresholds in non-dependent rats [32, 34, 41, 42, 43, 86]. In fact, even higher doses of mecamylamine alone (3.4–4.0 mg/kg) did not influence baseline ICSS thresholds [41, 87]. A non-specific effect of this dose of mecamylamine on ICSS thresholds is also incompatible with mecamylamine’s lack of effects on thresholds on test day 1 in the Nic + Mec ALL group (see Fig 2A).

Mecamylamine-precipitated elevations in ICSS thresholds in Experiment 1 were accompanied by increases in response latencies, suggesting a disruption of general motor function (see Methods for information on calculation and interpretation of response latency data). However, it is highly unlikely that mecamylamine’s motoric effects impacted its effects on ICSS thresholds. The ICSS threshold procedure used in this study is minimally impacted by response rate, as numerous treatments that alter ICSS latencies have little or no effect on ICSS thresholds [26, 35, 88]. We also found no correlation between mecamylamine-induced elevations in ICSS thresholds and response latencies within the same animals in Experiment 1, suggesting that mecamylamine’s effects on reinforcement sensitivity and motor function were independent.

In conclusion, our data show that a two-day continuous nicotine infusion is sufficient to demonstrate nicotine withdrawal in rats as measured by elevations in ICSS thresholds, with magnitude of these effects similar to those observed in traditional models involving more prolonged nicotine exposure prior to withdrawal testing. Further development of these models, including evaluation of potential moderating factors including nicotine exposure conditions (e.g., daily nicotine dose), subject characteristics (e.g., age, gender), and environmental variables (e.g., stress), could be useful for understanding the development of the nicotine withdrawal syndrome. Extending our findings to measures that simulate other aspects of nicotine withdrawal such as anxiety (e.g., elevated plus maze, open-field thigmotaxis), cognitive effects (e.g., contextual fear conditioning), and somatic signs also represents an important area for further research. Such work could help guide nicotine withdrawal-related research and treatment, and may also provide valuable scientific information for informing FDA regulation of tobacco products.

## Supporting Information

S1 Table Relationship between ICSS thresholds and response latencies following mecamylamine administration. Correlation coefficients between ICSS thresholds and ICSS response latencies on each test day in Experiment 1. The p-value for each correlation is italicized and in parentheses. Blank cells indicate that animals were not administered mecamylamine on that test day.
(DOCX)

S2 Table Effects of cessation of a 2-day or 9-day continuous nicotine infusion on ICSS latencies. ICSS response latencies (expressed as percent of baseline, mean ± SEM) during test sessions in Experiment 2.
(DOCX)
