# Supplementary material for: A Two-Day Continuous Nicotine Infusion Is Sufficient to Demonstrate Nicotine Withdrawal in Rats as Measured Using Intracranial Self-Stimulation
Source: PLoS One. 2015 Dec 11;10(12):e0144553. doi: 10.1371/journal.pone.0144553 (PMC4684239; doi:10.1371/journal.pone.0144553)
Supplement: S1 Table — Correlation coefficients between ICSS thresholds and ICSS response latencies on each test day in Experiment 1. The p-value for each correlation is italicized and in parentheses. Blank cells indicate that animals were not administered mecamylamine on that test day. (DOCX) [file pone.0144553.s001.docx]

|  | **Test Day** | | | | | | | | | |
| --- | --- | --- | --- | --- | --- | --- | --- | --- | --- | --- |
|  | **1** | **2** | **3** | **4** | **5** | **6** | **7** | **8** | **9** | **10** |
| **Nic + Mec ALL** | 0.20 (*0.71*) | 0.64 (*0.17*) | 0.14 (*0.80*) | 0.74 (*0.09*) | 0.51 (*0.30)* | 0.71 (*0.11*) | 0.40 (*0.44)* | 0.26 (*0.61*) | 0.71 (*0.11)* | 0.18 (*0.73)* |
| **Nic + Mec EVEN** |  | 0.31 (*0.49*) |  | 0.33 (*0.48*) |  | 0.33 (*0.47*) |  | 0.23 (*0.61*) |  | -0.41 (*0.36)* |
| **Nic + Mec FINAL** |  |  |  |  |  |  |  |  |  | -0.15 (*0.70)* |
