# Supplementary material for: A Two-Day Continuous Nicotine Infusion Is Sufficient to Demonstrate Nicotine Withdrawal in Rats as Measured Using Intracranial Self-Stimulation
Source: PLoS One. 2015 Dec 11;10(12):e0144553. doi: 10.1371/journal.pone.0144553 (PMC4684239; doi:10.1371/journal.pone.0144553)
Supplement: S2 Table — ICSS response latencies (expressed as percent of baseline, mean ± SEM) during test sessions in Experiment 2. (DOCX) [file pone.0144553.s002.docx]

|  | **Hours After Pump Removal** | | |
| --- | --- | --- | --- |
|  | **20** | **44** | **166** |
| **2-day Nic** | 94.8 ± 19.3 | 114.3 ± 8.1 | 104.1 ± 4.2 |
| **2-day Sal** | 99.4 ± 2.8 | 97.5 ± 2.5 | 99.0 ± 1.7 |
| **9-day Nic** | 119.0 ± 5.8 | 103.1 ± 8.1 | 94.7 ± 7.6 |
| **9-day Sal** | 101.3 ± 2.1 | 100.0 ± 1.9 | 103.7 ± 2.5 |
